# Identification of the Critical Sites of NNRTI-Resistance in Reverse Transcriptase of HIV-1 CRF_BC Strains

**DOI:** 10.1371/journal.pone.0093804

**Published:** 2014-04-17

**Authors:** Yang Huang, Zhenpeng Li, Hui Xing, Yang Jiao, Yabo Ouyang, Lingjie Liao, Shibo Jiang, Rebecca Armstrong, Yiming Shao, Liying Ma

**Affiliations:** 1 State Key Laboratory for Infection Disease Prevention and Control, National Center for AIDS/STD Control and Prevention (NCAIDS), Collaborative Innovation Center for Diagnosis and Treatment of Infectious Diseases, Chinese Center for Disease Control and Prevention (China-CDC), Beijing, China; 2 Key Laboratory of Medical Molecular Virology (Ministries of Education and Health), Shanghai Medical College and Institute of Medical Microbiology, Fudan University, Shanghai, China; Chinese Academy of Medical Sciences, China

## Abstract

**Background:**

The polymorphisms involved in drug resistance to non-nucleoside reverse transcriptase inhibitors (NNRTIs) in HIV-1 CRF_BC, the most prevalent HIV-1 strain in China, have been poorly characterized.

**Results:**

To reveal the drug resistance mutations, we compared the gene sequences of *pol* region of HIV-1 CRF_BC from 631 treatment-naïve and 363 treatment-experienced patients using the selection pressure-based method. We calculated an individual Ka/Ks value for each specific amino acid mutation. Result showed that eight polymorphic mutations (W88C, K101Q, I132L, R135L, T139K/R, H221Y and L228R) in RT for treatment-experienced patients were identified, while they, except for R135L, were completely absent in those from treatment-naïve patients. The I132L and T139K/R mutants exhibited high-level resistance to DLV and NVP and moderate resistance to TMC-125 and EFV, while the K101Q and H221Y mutants exhibited an increased resistance to all four NNRTIs tested. The W88C, R135L, and L228R may be RTI-induced adaptive mutations. Y181C+K101Q mutant showed a 2.5-, 4.4-, and 4.7-fold higher resistance to TMC-125, NVP and EFV, respectively, than Y181C alone mutant, while Y181C+H221Y or K103N+H221Y mutants had significantly higher resistance to all four NNRTIs than Y181C or K103N mutants. K103N+T139K and G190A+T139K mutant induce higher resistance (2.0∼14.2-fold and 1.5∼7.2-fold, respectively) to all four NNRTIs than K103N or G190A alone mutation.

**Conclusions:**

I132L and T139K/R are rare but critical mutations associated with NNRTI-resistance for some NNRTIs. K101Q, H221Y and T139K can enhance K103N/Y181C/G190A-assocated NNRTI-resistance. Monitoring these mutations will provide useful information for rational design of the NNRTI-based antiretroviral regimen for HIV-1 CRF_BC-infected patients.

## Introduction

Human immunodeficiency virus type 1 (HIV-1) has been categorized into nine genetically distinct subtypes within the M group, including subtypes A, B, C, D, F, G, H, J, and K. Recombination between genomes of two viruses of different subtypes results in generation of a circulating recombinant form (CRF) [1]. The distribution of these subtypes and CRFs varies widely by region. HIV-1 CRF_BC recombinant that was derived from subtype B′ (Thailand B) and Indian subtype C lineages has resulted in epidemics among the injecting drug users (IDUs) in China since this recombinant was first reported in 1999 [2], [3]. Currently, CRF_BC, which has been found in most parts of China, has become one of the most commonly transmitted HIV-1 subtypes across the country and was also found in other countries [4].

Rapid evolution and high mutation rate of HIV allow the virus to gain the ability of drug resistance. It is possible that HIV-1 genetic diversity may influence the type of resistance mutations that might eventually emerge upon drug exposure as well as the rate of emergence of resistance [5], [6]. Most studies have focused on the mechanisms of drug resistance of the subtype B viruses, which comprise only about 12% of HIV-1 cases in the world [7]. The currently available reverse transcriptase inhibitors have been widely used in the world, including China, against both B and non-B HIV-1 strains; however, the polymorphisms involving in drug resistance to non-nucleoside reverse transcriptase inhibitors (NNRTIs) in HIV-1 CRF_BC *pol* region have been poorly characterized. Particularly, the mutation sites associated with NNRTI-resistance in RT of HIV-1 CRF_BC viruses have not been reported [6].

In the present study, we compared the gene sequence of *pol* region of HIV-1 CRF_BC isolated from treatment–naïve and experienced patients, and then conducted the selection pressure analysis to identify rare but critical sites of mutations potentially associated with NNRTI-resistance. The association was further confirmed by using infectious clones with or without the newly identified mutations.

## Results

### Characteristics of the study populations

This study involved 994 HIV-1-positive patients, including 631 treatment-naïve patients (female: 29.6%; heterosexual contacts: 8.4%; intravenous drug use: 26.5%; unknown: 65.1%) and 363 ART-treated patients (female: 26.2%; heterosexual contacts: 19.8%; intravenous drug use: 29.2%; unknown: 51.0%). All the patients were identified to be infected by HIV-1 CRF_BC as determined by Neighbor-joining genetic analysis of *pol* sequences of the viruses obtained from plasma samples of the HIV-1-infected patients using PCR technique. The ART-experienced patients were receiving highly active antiretroviral therapy, including 2 NRTIs and 1 NNRTI. The NRTIs are lamivudine(3TC) plus zidovudine(AZT) or stavudine(d4T), while the NNRTI is either nevirapine(NVP) or efavirenz(EFV). Specifically, 13.5% of the patients had been treated with 3TC/AZT/EFV, 6.1% with 3TC/d4T/EFV, 58.7% with 3TC/AZT/NVP, 15.7% with 3TC/d4T/NVP, and 6.1% with unknown regimen. The mean treatment time was 18 months, including 28.0% for 0–6 months, 11.0% for 7–12 months, 23.1% for 13–18 months, 13.5% for 19–24 months, 17.9% for >24 months and 6.1% for unknown time.

### Polymorphism analysis of *pol* gene region of HIV-1 CRF_BC from plasmas of treatment-naïve and treatment-experienced patients

We used the selection pressure-based method, an important way to explore the rare but critical sites of drug resistance [10], [14]–[16], to investigate the association of these mutations with the drug resistance based on the criteria: (1) the Ka/Ks (the ratio of the number of non-synonymous substitutions per non-synonymous site (Ka) to the number of synonymous substitutions per synonymous site (Ks) and LOD (log odds ratio) value (confidence score to evaluate the significance of mutation or mutation pair) of the mutation in treatment samples was greater than 1 and 2, respectively, and the Ka/Ks of the mutation in treatment samples were larger than that in treatment-naïve samples; (2) Frequency of mutations in treatment was significantly larger than that in treatment-naive samples; (3) the non-synonymous mutations with low frequency (<1% treatment samples) were excluded. By evaluating the first 330 amino acids in HIV-1 RT sequences (the similarity of RT amino acids 1–330 between subtype B pNL4-3 and CRF_BC is 94.3%), we found that the frequencies of 15 polymorphism sites in RT of CRF_BC strains isolated from the treatment-experienced patients were significantly different from those isolated from the treatment-naïve patients (Table 1). In addition to the three previously reported RTI resistance-related mutations (A98G, Y188L, and G190A) [17], seven polymorphic mutations at seven positions (W88C, K101Q, I132L, T139K/R, H221Y and L228R) were presented in RT of CRF_BC strains isolated from the treatment-experienced patients, while they were completely absent in the RT of CRF_BC strains isolated from ART-naïve patients. Several mutations, including R135L, V179D, Y181C, M184V, K103N, were also present in the treatment-naïve patients who were infected by HIV-1 CRF_BC strains, but their frequencies were significantly increased in ART-treated group (P<0.01), while R135L isn't reported to be associated with drug resistance. In order to ascertain these polymorphism sites selected by NVP or EFV, the frequency of these mutations in patients with regimen containing NVP or EFV was compared. Of these mutations, A98G, T139R and L228R were solely selected by NVP, and Y181C has significantly higher frequency in NVP group than EFV group (*P* = 0.0012, fisher exact test).

**Table 1:** The changes of significant mutations between treatment-naïve and experienced patients.

Mutations^a^^,^ b	Treatment-naïve patients			Treatment-experienced patients			*P value* c
	Frequency (n)	Ka/Ks	LOD	Frequency (n)	Ka/Ks	LOD	
**W88C**	0.00% (0)	-	-	2.20% (8)	8.00	7.74	<0.001
A98G	0.00% (0)	-	-	1.93% (7)	26.82	6.38	<0.001
**K101Q**	0.00% (0)	-	-	1.65% (6)	4.38	2.64	0.001
K103N	0.95% (6)	1.85	2.23	18.73% (68)	32.29	64.51	<0.001
**I132L**	0.00% (0)	-	-	1.65% (6)	6.00	6.02	0.001
**R135L**	0.32% (2)	0.67	>2.00	3.31% (12)	12.00	>2.00	<0.001
**T139K**	0.00% (0)	-	-	2.20% (8)	13.14	6.62	<0.001
**T139R**	0.00% (0)	-	-	1.10% (4)	6.57	2.33	0.008
V179D	1.43% (9)	1.00	1.84	4.68% (17)	3.76	9.45	0.002
Y181C	0.63% (4)	0.18	0.02	10.47% (38)	4.22	13.37	<0.001
M184V	0.16% (1)	1.00	0.31	20.39% (74)	74.00	37.00	<0.001
Y188L	0.00% (0)	-	-	1.38% (5)	1.25	>2.00	0.003
G190A	0.00% (0)	-	-	7.16% (26)	11.07	24.78	<0.001
**H221Y**	0.00% (0)	-	-	6.61% (24)	2.40	6.65	<0.001
**L228R**	0.00% (0)	-	-	1.65% (6)	3.00	3.71	0.001

Note:

a The reference strain is CRF_BC.CN.CN54. The mutations listed denote the reference amino acid from HIV-1 subtype CRF07_BC.

b The bold mutations are those that have not been reported to be associated with drug resistance.

c *P*-value was computed by using chi-square test.

### Susceptibility to NNRTIs against HIV-1 CRF_BC strains with the newly identified mutations in RT

To investigate the contribution of these mutations to NNRTI resistance, the sensitivities of the viruses with WT and MT in RT to each NNRTI used, including Etravirine (TMC-125), Rescriptor (DLV), Viramune (NVP), and Sustiva (EFV), were determined. CRF_BC strains with K103N and Y181C in RT were included as controls. The K101Q, I132L and T139K/R mutants exhibited significant (2∼28-fold) increases in resistance to all the four NNRTIs tested (*P*<0.05), and H221Y mutant had a moderate increase (approximately 2-fold) of resistance to these four NNRTIs (*P*<0.05), while the W88C, R135L and L228R mutations had no significant effect on the viral resistance to RTIs (Table 2). Besides K101Q and H221Y, the other three mutants I132L and T139K/R were rarely reported to associate with drug resistance. We found that HIV-1 subtype B viruses with I132L and T139K/R mutations were also resistant to NNRTIs, although their resistant level is relatively lower than that of HIV-1 CRF_BC viruses with these mutations (Table 2).

**Table 2:** Sensitivity and resistance of different mutation sites in HIV-1 CRF_BC *pol* region to NNRTIs using an *in vitro* phenotypic assay^a^.

Mutations	TMC-125		DLV		NVP		EFV	
	EC50^a^ (nM)	Fold change^c^	EC50 (µM)	Fold change	EC50 (µM)	Fold change	EC50 (nM)	Fold change
WT^b^ (BC)	1.03±0.10	-	0.09±0.00	-	0.10±0.01	-	1.713±0.180	-
**W88C** (BC)	1.09±0.02	1.06	0.05±0.00	0.51	0.09±0.01	0.89	0.98±0.06	0.57
**K101Q** (BC)	3.61±0.35	3.50	0.15±0.01	1.71	1.30±0.14	12.57	5.25±1.80	3.06
K103N (BC)	1.400±0.06	1.35	7.35±0.23	82.56	17.23±1.36	167.23	97.48±4.06	56.91
**I132L** (BC)	5.21±0.78	2.55	1.72±0.12	19.35	4.30±0.26	28.05	10.52±1.22	6.13
**R135L** (BC)	1.36±0.15	1.32	0.07±0.00	0.75	0.12±0.00	1.13	1.87±0.13	1.09
**T139R** (BC)	3.73±0.62	1.82	0.42±0.02	4.69	2.59±0.53	25.12	3.23±0.38	1.89
**T139K** (BC)	4.82±0.96	4.67	0.33±0.02	3.66	0.76±0.02	7.35	5.65±0.12	3.30
M184V (BC)	0.69±0.04	0.67	0.03±0.00	0.31	0.05±0.01	0.44	0.70±0.05	0.41
Y181C (BC)	5.91±1.700	5.73	4.53±0.13	50.92	15.84±1.42	153.74	3.22±0.14	1.88
**H221Y** (BC)	1.42±0.16	1.38	0.18±0.01	2.04	0.21±0.03	2.02	3.02±0.51	1.76
**L228R** (BC)	1.02±0.101	0.99	0.042±0.001	0.47	0.11±0.00	1.03	0.79±0.04	0.46
WT^b^ (B)	1.66±0.35	-	0.11±0.00	-	0.19±0.01	-	3.26±0.25	-
**I132L** (B)	6.51±1.19	3.91	0.51±0.02	4.61	1.20±0.03	6.38	11.61±0.19	3.56
**T139K** (B)	5.21±1.03	3.13	0.200±0.01	1.78	0.43±0.03	2.26	6.87±0.79	2.10
**T139R** (B)	9.64±0.87	5.79	0.44±0.01	3.99	1.09±0.05	5.78	8.97±0.63	2.75

Note:

a The unit for the EC50 values of TMC-125 and EFV is nM, while that for the EC50 values of DLV and NVP is µM. Data were presented as the mean ± standard deviations of three separate determinations.

b WT: HIV-1 CRF_BC strain N-14-1.

c Fold change was determined by calculating the ratio of EC50s for mutations and WT viruses.

### Characterizing the mutation relationship based on predicted drug resistance mutations interaction network

To determine the influence of the mutation of one site to another, a conditional selection ratio was computed. If the conditional selection ratio of X to Y (X→Y) is greater than 1 and LOD is greater than 2, the influence of X to Y (X→Y) was considered significant. Then, software Cytoscape was used to construct the relationship among these predicted drug resistance mutations as reported [18].

**Figure 1:**
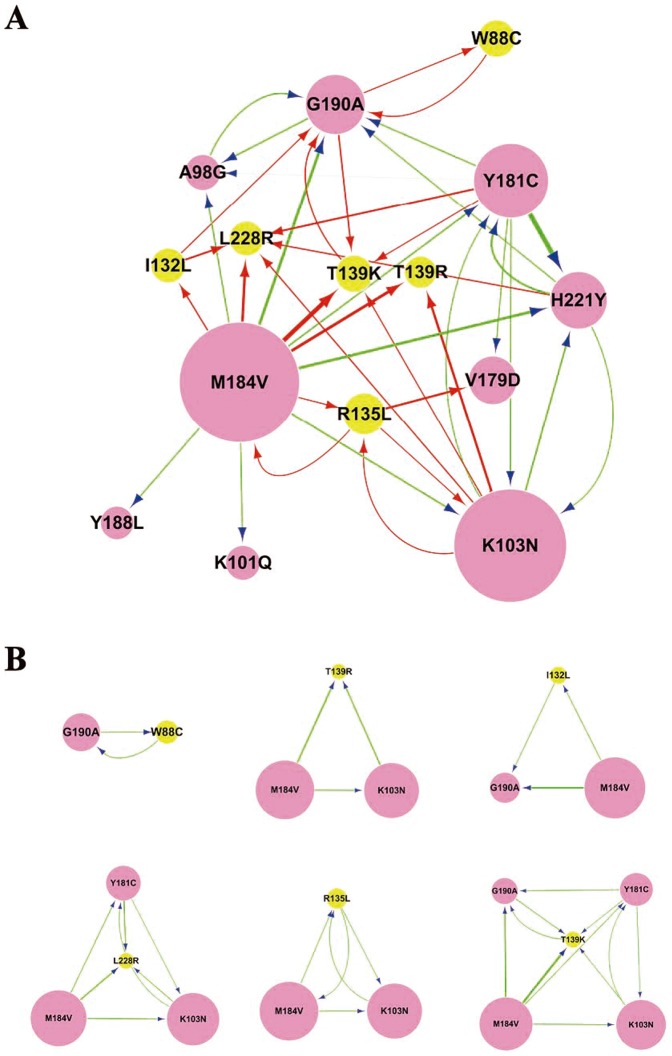
Predicted interaction network of NNRTI-resistance related mutations. The network in (A) represents the global relationship among the potential NNRTI-resistance related mutations, while (B) shows the relationship between a rare but critical mutation and the well-known RTI-resistance mutations. The rare but critical mutations are highlighted in yellow, and the arrows from the source node to the target node indicate the influence of one site on another. In the network, the size of the node represents the mutation frequency of that site from one amino acid to another, while the width of line represents the strength of influence between two mutations.

The network represents the comprehensive relationship among the predicted drug-resistance mutations, and the arrows from the source node to the target node indicate the influence of one to another. In the network, the size of the node represents the mutation frequency of that site from one amino acid to another, while the width of line represents the influence strength between two mutations. As shown in Figure 1, the network contained 15 mutation sites which have 40 interaction relationships (Table S1). In the network, mutations with higher frequency, such as M184V and K103N, were more likely to influence the other mutations. For example, M184V and K103N had 12 (A98G, K101Q, K103N, I132L, R135L, T139K, T139R, Y181C, Y188L, G190A, H221Y, and L228R) and 6 (R135L, T139K, T139R, Y181C, H221Y, L228R) target mutations, respectively.

The mutation T139K may be induced by other mutations, including K103N, Y181C, M184V and G190A, and the selection pressure ratio from G190A to T139K reach to 7. Notably, T139K mutation had a significant influence on G190A, indicating a correlation between the two mutations. The mutual influence between T139K and G190A hints that these two mutations may form as a mutation pattern to function synergistically. Interestingly, H221Y was associated with Y181C and/or K103N mutations. For example, K103N, Y181C and H221Y are three mutations formed by pairwise interactions. Y181C and H221Y, in particular, have strong mutual influence (conditional selection ratio of Y181C→H221Y and H221Y→Y181C was 45 and 11, respectively), suggesting that H221Y and Y181C may form combinatorial mutation patterns to synergistically resist the drug treatment.

### Susceptibility to NNRTIs of HIV-1 CRF_BC strains with the newly identified in combination with the well-known Y181C, G190A or K103N mutation

We next examined the effect of the single mutation sites listed above in combination with Y181C or K103N on viral resistance to NNRTIs. The pNL4-3 clone containing HIV-1CRF_BC *pol* with mutations Y181C, G190A or K103N was constructed through site-directed mutagenesis with or without the newly identified mutations in this study. We tested the phenotypic resistance of these combinations Y181C, G190A or K103N with different mutation sites in RT of HIV-1 CRF_BC to NNRTIs using an *in vitro* phenotypic assay. As shown in Table 3, Y181C+K101Q mutant showed a 2.48-, 4.37-, and 4.69-fold higher resistance to TMC-125, NVP and EFV, respectively, than Y181C alone mutant (*P*<0.05). Y181C+H221Y mutations resulted in significantly higher resistance to all four NNRTIs than Y181C alone mutation, ranging in 3.00∼4.24 FC (*P*<0.05). K103N+T139K mutant induce higher resistance to all four NNRTIs, with FC ranging from 2.00 to 14.15. K103N+H221Y mutations exhibited an increased (1.69- to 2.96-fold) resistance to the four NNRTIs tested (*P*<0.05), while K103N+K101Q mutants did not displayed a higher NNRTI-resistance than K103N alone mutant. G190A+T139K also showed a higher increased (1.48- to 7.21-fold) resistance to all four NNRTIs than G190A alone mutation.

**Table 3:** Phenotypic resistance of Y181C or K103N combined with different mutation sites in HIV-1 CRF_BC pol region to NNRTIs using an *in vitro* phenotypic assay.

Mutations	TMC-125		DLV		NVP		EFV	
	EC50^a^ (nM)	Fold change^b^	EC50 (µM)	Fold change	EC50 (µM)	Fold change	EC50 (µM)	Fold change
Y181C	5.91±1.70	-	4.53±0.13	-	13.95±0.44	-	3.22±0.14	-
Y181C+K101Q	14.67±0.36	2.48	7.26±0.24	1.60	60.95±2.42	4.37	15.11±0.18	4.69
Y181C+H221Y	19.19±0.29	3.25	13.60±0.82	3.00	59.17±8.93	4.24	11.76±1.67	3.65
Y181C+L228R	6.34±0.92	1.07	3.50±0.22	0.77	26.35±1.88	1.89	3.37±0.55	1.05
K103N	1.40±0.06	-	7.35±0.23	-	17.23±1.36	-	97.48±4.06	-
K103N+K101Q	2.14±0.16	1.53	7.07±0.48	0.96	25.20±0.52	1.46	124.19±14.47	1.28
K103N+H221Y	3.96±0.46	2.83	21.75±0.84	2.96	30.85±0.49	1.79	164.41±9.02	1.69
K103N++L228R	1.78±0.18	1.28	5.23±0.16	0.71	17.60±0.97	1.02	153.94±1.10	1.59
K103N+T139K	2.80±0.14	2.00	>100.00	>14.15	82.08±8.20	4.77	408.95±52.69	4.20
G190A	1.81±0.53		0.02±0.01		6.11±0.13		8.31±0.72	
G190A +T139K	2.92±0.44	1.61	0.03±0.00	1.48	44.01±1.70	7.21	15.68±1.45	1.87

Note:

a The unit for the EC50 values of TMC-125 is nM, while that for the EC50 values of DLV, NVP and EFV is µM. Data were presented as the mean ± standard deviations of three separate determination.

b Fold change was determined by calculating the ratio of EC50s for mutations and WT viruses.

Each sample was tested in triplicate, and each experiment was repeated twice. EC50 (mM, except nM for maraviroc) data are presented as means 6 standard deviations.

## Discussion

Most of the current anti-HIV drugs have not been tested in the clinical trials in China, drawing attention to the effectiveness of these drugs against the HIV-1 strains circulation in China. We recently have shown that Fuzeon and Maraviroc, the only two HIV entry inhibitors approved for clinical use by the US FDA, are much less effective against the HIV-1 subtypes circulating in China than the B subtype predominating in the United States and Europe [5]. Therefore, it is essential to study the effectiveness of a new class of antiretroviral drugs, such as NNRTIs, before they are introduced into China.

At present, the antiretroviral drugs have been used not only for treatment, but also for prevention of HIV infection/AIDS [19]. HIV clinical trials revealed the magnitude of benefit when using antiretroviral drugs to prevent sexual transmission or mother-to-child transmission of HIV-1 [20], [21], suggesting the new use of antiretroviral drugs for pre- and post-exposure prophylaxis [22]. Therefore, analysis of the drug-resistance becomes more and more important for rational design of therapeutic and prophylactic regimen.

Some *in vitro* and *in vivo* observations suggest that the various subtypes may respond differently to NNRTIs [23]. The frequency and pattern of mutations conferring resistance to these drugs differ among HIV-1 subtypes and can influence the outcome [24]. CRF_BC strain accounted for more than half of HIV-1 infection in China [25]. As a result, it is particularly important to understand the mutation changes between ART-naïve and ART-experienced patients infected by CRF_BC and their effect on dug-resistance.

By using the selection pressure-based method, we compared the gene sequences of *pol* region of HIV-1 strains isolated from 631 treatment-naïve patients and 363 ART-treated patients who were verified to be infected by HIV-1 CRF_BC. We found that the frequencies of 15 polymorphism sites in RT of CRF_BC strains isolated from the treatment-experienced patients were significantly different from those isolated from the treatment-naïve patients. Especially, seven mutations at six positions (W88C, K101Q, I132L, R135L, T139K/R, H221Y and L228R) were completely absent in the RT of CRF_BC strains isolated from drug-naïve patients. In contrast, their frequencies in strains isolated from ART-treated patients were significantly increased, suggesting their specific association with ART treatment. Since the ART regimen of these patients contained two NRTIs and one NNRTI, *in vitro* experiments were tested for susceptibility to 3TC, d4T, AZT, TFV. The results demonstrated that these mutations were not associated with the resistance to NRTIs (Table S2), We postulate that these mutations may have effect on their sensitivity to NNRTIs. Five mutants (K101Q, I132L, T139K/R and H221Y) among these eight mutants exhibited an increased resistance to the four NNRTIs tested. According to Stanford HIV resistance database, the mutations of I132L, T139K and T139R were rare events (0.11%, 0.57% and 4%, respectively) in B subtype under treatment, which may indicate the higher genetic barrier for these three mutations in B subtype than CRF_BC. Although it is reported that K101Q and H221Y may belong to the ETR RAMs [26], and H221Y was a mutation responsible for drug-resistance to Rilpivirine [27], our study has shown for the first time that both K101Q and H221Y mutations are associated with the increased resistance to all the four NNRTIs tested. Our study has demonstrated that the viruses with I132L and T139K/R mutations that exhibited high-level resistance to NNRTIs are the rare but critical mutants associated with NNRTI-resistance in both CRF_BC and B subtype.

The potential mechanistic association between the NNRTI-resistance and the I132L and T139K/R mutations may be ascribed to the location of these mutation sites. All of the three mutations are located in the β7/β8 loop (residues 132–140) of RT, which is involved in the formation of the base of the NNRTI-binding pocket [28], [29]. Mutations of these residues may cause the conformation change of the pocket, resulting in the decreased binding between the NNRTI and the pocket in RT. It was also reported that T139K mutation could seriously impair catalytic activities of RT [30].

The increasing evidences suggest that in addition to those currently known mutations, more and more unidentified mutations may also be involved in the development of NNRTI resistance, which contribute to NNRTI therapy failure [6], and the development of resistance to NNRTIs may be more complex than the classical one-step model of significant resistance via a single mutation so far considered [31]. It has been reported that HIV can employ various combinations of mutations to resist drug treatments [32]. To further determine mutational interactions between the newly identified and unknown mutations in RT of CRF_BC strains, a conditional selection ratio were computed. We found that all mutations were connected together as a component and in the network, mutations of high frequency were more likely to influence the other mutations (Fig. 1). The relationship among mutations in the networks can give clues to the combinatorial mutation patterns responsible for HIV drug resistance within the network. Particularly, H221Y were associated with Y181C and/or K103N mutations in RT of CRF_BC strains isolated from the treatment-experienced patients and K101Q showed positive interaction with M184V. Others have also reported similar combinational mutations, although the effect of these combined mutations on drug-resistance has not been clearly defined [6], [33]. To understand the effect of our newly identified mutations combined with those known mutations, we examined the effect of the single mutation sites in combination with Y181C, G190A or K103N on viral resistance to NNRTIs. The result showed that either Y181C+H221Y or K103N+H221Y mutants exhibited significantly enhanced resistance to all the four NNRTIs tested, compared with Y181C alone and K103N alone mutants. Y181C+K101Q mutants also showed higher resistance to TMC-125, NVP and EFV than Y181C alone mutant. K103N+T139K and G190A+T139K mutants induce an increased resistance to all four NNRTIs. These results suggest that K101Q, T139K and H221Y are able to enhance the NNRTI-resistance mediated by those well-characterized HIV-1 mutants. The positive interaction between K101Q and M184V is of interest and will be investigated in vitro in future time.

In summary, our data suggest that I132L and T139K/R mutations that exhibited high-level resistance to NNRTIs are the rare but critical mutants associated with NNRTI-resistance in RT of CRF_BC strains that are predominantly circulating in China, while K101Q and H221Y mutations are associated with the increased resistance to all the four NNRTIs tested, although at codons 101 and 221 were reported relating to NNRTI resistance. The co-presence of H221Y, T139K or K101Q with the well-known RTI-resistance mutations K103N, G190A or Y181C may strengthen the drug-resistance effect. Further study is needed to determine how these mutations and combined mutations affect the binding kinetics of NNRTIs. We suggest that these newly identified mutations should be considered for the improvement of algorithms that predict clinical responses to antiretroviral drugs and for assessing the efficacies of next-generation drugs. This information will aid in designing initial treatment strategies for persons infected with CRF_BC viruses and interpreting genetic resistance among the CRF_BC-infected patients whose antiretroviral therapy has failed.

## Methods

### Study population

The study population included pre-selected HIV-1-positive patients with treatment-naïve and experienced antiretroviral therapies, who participated in a multicenter AIDS Cohort Study including China Global Fund AIDS Program, and “Eleven Five” major projects in Xinjiang and Sichuan provinces of China during 2007–2011. The individuals who newly HIV-infection screened and confirmed were investigated without experiencing ART were chosen as the treatment- naïve patients in Xinjiang and Sichuan province of China during that time. The HIV/AIDS patients who received ART with 2 NRTIs and 1 NNRTIs regimen in the two provinces were investigated to detect viral load and CD4 count periodically. When the patients encountered virological failure during ART according to WHO ARV therapy failure criteria (the virological failure was defined as a viral load of ≥10 000 copies/ml) [8], they were recruited as the treatment-experienced patients. To obtain the CRF_BC recombinant representative isolates, 994 patients were chosen through sequence blastx on the website (http://www.hiv.lanl.gov/content/sequence/BASIC_BLAST/basic_blast.html). Furthermore, to confirm these sequences, we conducted a Neighbor-joining genetic analysis of *pol* sequences obtained from plasma samples of all HIV-1-infected patients using the PCR technique as previously described [9]. This study was approved by the Institutional Research Ethics Community, China CDC, and all subjects signed informed consent forms before blood collection.

### HIV-1 *pol* sequence detection

HIV *pol* sequence was carried out by an in-house polymerase chain reaction protocol as previously described [9] Briefly, viral RNA was extracted from patient's plasma using a QIAamp Viral RNA Mini Kit (Qiagen Inc., Chatsworth, CA) and cDNA was generated using primer RT21 (CTGTATTTCAGCTATCAAGTCTTTTG ATGGG). A nested PCR was then employed using the generated cDNA as template. The nested PCR product was purified using a QIAquick Gel Extraction Kit (Qiagen Inc) and sequenced with the ABI 3100 DNA Sequencer.

### Ka/Ks and Conditional selection ratio calculation

The Ka/Ks values for specific amino acid substitutions were determined as described by Chen et al [10]. To measure how a specific amino acid of one site X influences one in the other site Y. The ‘conditional selection ratio’ is defined as the ratio of Ka/Ks of Y when the amino acid is mutated at X (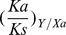) divided by the Ka/Ks of Y in the absence of any mutation at X (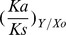), and it was computed as follows:
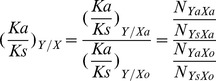
Where N_YaXa_ is the number of samples with the same amino acid mutation both at site Y and X; and N_YsXa_ is the number of samples with a synonymous mutation at codon Y and an amino acid mutation at codon X. N_YaXo_ and N_YsXo_ are the number of samples with the amino acid mutation and a synonymous mutation at codon Y in the absence of any mutation at X respectively.

The LOD score by which we evaluated the significance of apparent amino acid pairs was calculated using the following formula:
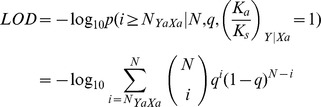
Where N = N_YaXa_+N_YsXa_ and q as defined above.

If LOD>2, the positive selection is significant.

### Construction of new pNL4-3 containing HIV-1 CRF_BC *pol* gene with site-directed mutagenesis

The infectious molecular clone was constructed by incorporating amplified PR and RT regions of CRF_BC into pNL4-3 using BstE II and Age I restriction sites after BstE II at position 2049 (RT region) of pNL4-3 was created by replacing A with T. HIV-1 CRF_BC (CBJB257), which was isolated from treatment-naïve intravenous drug user in Xinjiang, China [11], was chosen for viral DNA extraction by a QIAamp Viral DNA Mini Kit (Qiagen Inc., Chatsworth, CA). The extracted viral DNA was used as the template for first-round PCR as previously described [9]. The first-round PCR product and primers (GGAAGGTCACCAAATGAAAGATTGTACTGAGAG and TGTACCGGTTCTTTTAG AATCTCCCTGTTTTCTGCC) were used for second-round PCR, which underlined sequences mark the relevant restriction sites. The nested PCR product was purified using a QIAquick Gel Extraction Kit (Qiagen Inc), digested with BstE II and AgeI (NEB) and then ligated to BstE II - and AgeI-digested pNL4-3. The mutations were introduced into CBJB257 RT regions inserted in T-vector by using site-directed mutagenesis with DNA polymerase (PrimerStar, Takara) and site mutation primers. DNA sequencing was performed in both directions across the entire RT-coding region to verify the absence of spurious mutations and the presence of the desired mutation. It should be noted that the cloned fragment of CRF_BC RT encompass just about 300 aminos of N-terminus. Although the mutations of the other region in RT may enhance resistance to HIV drugs, such as some mutations in the connection domain, such situation should be ruled out because pNL4-3 was wild type reference strain without such mutations.

### Phenotypic assay to HIV-1 NNRTIs based on TZM-bl cells

HIV-1 (HIV-1WT) and HIV-1 with the mutations (HIV-1MT) were generated by transfection of the plasmids into 293T/17 cells by using Fugene 6 Transfection Reagent (Roche Applied Science) according to the manufacturer's instructions. The 50% tissue culture infectious dose (TCID_50_) and the antiviral activity of NNRTIs were determined using TZM-b1 cells as previously described [12], [13]. The concentration of drug that effects 50% viral replication (EC50) values was determined by nonlinear regression using GraphPad Prism 5.01. Mean EC50 were calculated using all replicates for each virus and are expressed as mean ± SD. The Wilcoxon rank sum test was applied to pairwise comparisons to determine whether the observed differences between EC50 for different site-mutations were statistically significant.

## Supporting Information

Table S1 **The Conditional selection ratio among drug resistance related mutations.**
(DOC)

Table S2 **Sensitivity and resistance of different mutation sites in HIV-1 CRF_BC RT region to NRTIs.**
(DOC)

## References

[1] Archer J, Robertson DL (2007) Understanding the diversification of HIV-1 groups M and O. AIDS 21: 1693–1700 10.1097/QAD.0b013e32825eabd0 17690566

[2] Shao Y, Zhao F, Yang W (1999) [The identification of recombinant HIV-1 strains in IDUs in southwest and northwest China]. Zhonghua Shi Yan He Lin Chuang Bing Du Xue Za Zhi 13: 109–112. 12569772

[3] Piyasirisilp S, McCutchan FE, Carr JK, Sanders-Buell E, Liu W, et al (2000) A recent outbreak of human immunodeficiency virus type 1 infection in southern China was initiated by two highly homogeneous, geographically separated strains, circulating recombinant form AE and a novel BC recombinant. J Virol 74: 11286–11295. 11070028 10.1128/jvi.74.23.11286-11295.2000 PMC113233

[4] Ouyang Y, Shao Y, Ma L (2012) HIV-1 CRF_BC recombinants infection in China: molecular epidemic and characterizations. Curr HIV Res 10: 151–161. 22329522 10.2174/157016212799937236

[5] Yu X, Yuan L, Huang Y, Xu W, Fang Z, et al (2011) Susceptibility of HIV-1 subtypes B', CRF07_BC and CRF01_AE that are predominantly circulating in China to HIV-1 entry inhibitors. PLoS ONE 6: e17605 10.1371/journal.pone.0017605 21412427 PMC3055885

[6] Ceccherini-Silberstein F, Svicher V, Sing T, Artese A, Santoro MM, et al (2007) Characterization and structural analysis of novel mutations in human immunodeficiency virus type 1 reverse transcriptase involved in the regulation of resistance to nonnucleoside inhibitors. J Virol 81: 11507–11519 10.1128/JVI.00303-07 17686836 PMC2045529

[7] Brenner BG (2007) Resistance and viral subtypes: how important are the differences and why do they occur? Curr Opin HIV AIDS 2: 94–102 10.1097/COH.0b013e32801682e2 19372873

[8] Keiser O, MacPhail P, Boulle A, Wood R, Schechter M, et al (2009) Accuracy of WHO CD4 cell count criteria for virological failure of antiretroviral therapy. Trop Med Int Health 14: 1220–1225 10.1111/j.1365-3156.2009.02338.x 19624478 PMC3722497

[9] Ma L, Huang J, Xing H, Yuan L, Yu X, et al (2010) Genotypic and phenotypic cross-drug resistance of harboring drug-resistant HIV type 1 subtype B' strains from former blood donors in central Chinese provinces. AIDS Res Hum Retroviruses 26: 1007–1013 10.1089/aid.2009.0252 20718629

[10] Chen L, Perlina A, Lee CJ (2004) Positive selection detection in 40,000 human immunodeficiency virus (HIV) type 1 sequences automatically identifies drug resistance and positive fitness mutations in HIV protease and reverse transcriptase. J Virol 78: 3722–3732. 15016892 10.1128/JVI.78.7.3722-3732.2004 PMC371046

[11] Ma L, Guo Y, Yuan L, Huang Y, Sun J, et al (2009) Phenotypic and genotypic characterization of human immunodeficiency virus type 1 CRF07_BC strains circulating in the Xinjiang Province of China. Retrovirology 6: 45 10.1186/1742-4690-6-45 19442296 PMC2693499

[12] Wei X, Decker JM, Liu H, Zhang Z, Arani RB, et al (2002) Emergence of resistant human immunodeficiency virus type 1 in patients receiving fusion inhibitor (T-20) monotherapy. Antimicrob Agents Chemother 46: 1896–1905. 12019106 10.1128/AAC.46.6.1896-1905.2002 PMC127242

[13] Xu H-T, Quan Y, Schader SM, Oliveira M, Bar-Magen T, et al (2010) The M230L nonnucleoside reverse transcriptase inhibitor resistance mutation in HIV-1 reverse transcriptase impairs enzymatic function and viral replicative capacity. Antimicrob Agents Chemother 54: 2401–2408 10.1128/AAC.01795-09 20308384 PMC2876396

[14] Qiu P, Sanfiorenzo V, Curry S, Guo Z, Liu S, et al (2009) Identification of HCV protease inhibitor resistance mutations by selection pressure-based method. Nucleic Acids Res 37: e74 10.1093/nar/gkp251 19395595 PMC2691846

[15] Chen L, Lee C (2006) Distinguishing HIV-1 drug resistance, accessory, and viral fitness mutations using conditional selection pressure analysis of treated versus untreated patient samples. Biol Direct 1: 14 10.1186/1745-6150-1-14 16737543 PMC1523337

[16] Pan C, Kim J, Chen L, Wang Q, Lee C (2007) The HIV positive selection mutation database. Nucleic Acids Res 35: D371–375 10.1093/nar/gkl855 17108357 PMC1669717

[17] Johnson VA, Calvez V, Günthard HF, Paredes R, Pillay D, et al (2011) 2011 update of the drug resistance mutations in HIV-1. Top Antivir Med 19: 156–164. 22156218 PMC6148877

[18] Shannon P, Markiel A, Ozier O, Baliga NS, Wang JT, et al (2003) Cytoscape: a software environment for integrated models of biomolecular interaction networks. Genome Res 13: 2498–2504 10.1101/gr.1239303 14597658 PMC403769

[19] Cohen MS, Chen YQ, McCauley M, Gamble T, Hosseinipour MC, et al (2011) Prevention of HIV-1 infection with early antiretroviral therapy. N Engl J Med 365: 493–505 10.1056/NEJMoa1105243 21767103 PMC3200068

[20] Coovadia HM, Brown ER, Fowler MG, Chipato T, Moodley D, et al (2012) Efficacy and safety of an extended nevirapine regimen in infant children of breastfeeding mothers with HIV-1 infection for prevention of postnatal HIV-1 transmission (HPTN 046): a randomised, double-blind, placebo-controlled trial. Lancet 379: 221–228 10.1016/S0140-6736(11)61653-X 22196945 PMC3539769

[21] Kashuba ADM, Patterson KB, Dumond JB, Cohen MS (2012) Pre-exposure prophylaxis for HIV prevention: how to predict success. Lancet 379: 2409–2411 10.1016/S0140-6736(11)61852-7 22153566 PMC3652584

[22] Liu AY, Grant RM, Buchbinder SP (2006) Preexposure prophylaxis for HIV: unproven promise and potential pitfalls. JAMA 296: 863–865 10.1001/jama.296.7.863 16905792

[23] Lai M-T, Lu M, Felock PJ, Hrin RC, Wang Y-J, et al (2010) Distinct mutation pathways of non-subtype B HIV-1 during in vitro resistance selection with nonnucleoside reverse transcriptase inhibitors. Antimicrob Agents Chemother 54: 4812–4824 10.1128/AAC.00829-10 20805392 PMC2976110

[24] Holguín A, Ramirez de Arellano E, Rivas P, Soriano V (2006) Efficacy of antiretroviral therapy in individuals infected with HIV-1 non-B subtypes. AIDS Rev 8: 98–107. 16848277

[25] Liao L, Xing H, Dong Y, Qin G, Ma Y, et al (2012) Surveys of transmitted HIV drug resistance in 7 geographic Regions in China, 2008–2009. Clin Infect Dis 54 Suppl 4: S320–323 10.1093/cid/cir1016 22544196

[26] Asahchop EL, Oliveira M, Wainberg MA, Brenner BG, Moisi D, et al (2011) Characterization of the E138K resistance mutation in HIV-1 reverse transcriptase conferring susceptibility to etravirine in B and non-B HIV-1 subtypes. Antimicrob Agents Chemother 55: 600–607 10.1128/AAC.01192-10 21135184 PMC3028807

[27] Azijn H, Tirry I, Vingerhoets J, de Béthune M-P, Kraus G, et al (2010) TMC278, a next-generation nonnucleoside reverse transcriptase inhibitor (NNRTI), active against wild-type and NNRTI-resistant HIV-1. Antimicrob Agents Chemother 54: 718–727 10.1128/AAC.00986-09 19933797 PMC2812151

[28] Pandey PK, Kaushik N, Talele TT, Yadav PN, Pandey VN (2001) The beta7-beta8 loop of the p51 subunit in the heterodimeric (p66/p51) human immunodeficiency virus type 1 reverse transcriptase is essential for the catalytic function of the p66 subunit. Biochemistry 40: 9505–9512. 11583149 10.1021/bi002872j

[29] Pandey PK, Kaushik N, Singh K, Sharma B, Upadhyay AK, et al (2002) Insertion of a small peptide of six amino acids into the beta7-beta8 loop of the p51 subunit of HIV-1 reverse transcriptase perturbs the heterodimer and affects its activities. BMC Biochem 3: 18. 12086585 10.1186/1471-2091-3-18 PMC117134

[30] Auwerx J, Rodríguez-Barrios F, Ceccherini-Silberstein F, San-Félix A, Velázquez S, et al (2005) The role of Thr139 in the human immunodeficiency virus type 1 reverse transcriptase sensitivity to (+)-calanolide A. Mol Pharmacol 68: 652–659 10.1124/mol.105.012351 15961674

[31] Bacheler LT, Anton ED, Kudish P, Baker D, Bunville J, et al (2000) Human immunodeficiency virus type 1 mutations selected in patients failing efavirenz combination therapy. Antimicrob Agents Chemother 44: 2475–2484. 10952598 10.1128/aac.44.9.2475-2484.2000 PMC90088

[32] Zhang J, Hou T, Wang W, Liu JS (2010) Detecting and understanding combinatorial mutation patterns responsible for HIV drug resistance. Proc Natl Acad Sci USA 107: 1321–1326 10.1073/pnas.0907304107 20080674 PMC2824344

[33] Kulkarni R, Babaoglu K, Lansdon EB, Rimsky L, Van Eygen V, et al (2012) The HIV-1 reverse transcriptase M184I mutation enhances the E138K-associated resistance to rilpivirine and decreases viral fitness. J Acquir Immune Defic Syndr 59: 47–54 10.1097/QAI.0b013e31823aca74 21997204

