# Supplementary material for: Identification of the Critical Sites of NNRTI-Resistance in Reverse Transcriptase of HIV-1 CRF_BC Strains
Source: PLoS One. 2014 Apr 17;9(4):e93804. doi: 10.1371/journal.pone.0093804 (PMC3990534; doi:10.1371/journal.pone.0093804)
Supplement: Table S1 — The Conditional selection ratio among drug resistance related mutations. (DOC) [file pone.0093804.s001.doc]

Table S1. The Conditional selection ratio among drug resistance related mutations

| Source | Target | Conditional selection ratio | LOD | Source | Target | Conditional selection ratio | LOD |
| --- | --- | --- | --- | --- | --- | --- | --- |
| G190A | W88C | 2.16 | 3.08 | K103N | Y181C | 1.46 | 4.03 |
| Y181C | A98G | 1.20 | 3.08 | M184V | Y181C | 6.46 | 11.20 |
| M184V | A98G | 4.00 | 6.15 | H221Y | Y181C | 10.59 | 11.20 |
| G190A | A98G | 6.00 | 6.15 | R135L | M184V | 1.80 | 5.04 |
| M184V | K101Q | 1.67 | 2.10 | M184V | Y188L | 4.50 | Inf* |
| R135L | K103N | 2.00 | 9.89 | W88C | G190A | 2.17 | 3.08 |
| Y181C | K103N | 1.61 | 11.35 | A98G | G190A | 4.55 | 5.46 |
| M184V | K103N | 7.11 | 35.63 | I132L | G190A | 3.52 | 4.61 |
| H221Y | K103N | 1.37 | 10.15 | T139K | G190A | 5.14 | 6.15 |
| M184V | I132L | 6.00 | 7.42 | Y181C | G190A | 4.41 | 7.80 |
| K103N | R135L | 2.67 | >2.00 | M184V | G190A | 20.57 | 24.59 |
| M184V | R135L | 3.00 | >2.00 | H221Y | G190A | 3.39 | 4.03 |
| K103N | T139K | 3.60 | 4.02 | K103N | H221Y | 7.50 | 5.04 |
| Y181C | T139K | 2.33 | 4.02 | Y181C | H221Y | 45.00 | 10.01 |
| M184V | T139K | 42.00 | 3.08 | M184V | H221Y | 20.00 | 9.10 |
| G190A | T139K | 7.00 | 9.87 | K103N | L228R | 5.33 | 3.89 |
| K103N | T139R | 18.00 | 6.15 | I132L | L228R | 11.00 | 3.08 |
| M184V | T139R | 24.00 | 5.46 | Y181C | L228R | 11.33 | 4.64 |
| R135L | V179D | 15.00 | 4.02 | M184V | L228R | 20.00 | 4.99 |
| Y181C | V179D | 1.34 | 2.47 | H221Y | L228R | 5.25 | 2.31 |

Note: 'Source' means the source mutation,

'Target' means the target mutation

'Conditional selection ratio' can be used as an index to measure how source mutation influence target mutation.
