# Supplementary material for: Identification of the Critical Sites of NNRTI-Resistance in Reverse Transcriptase of HIV-1 CRF_BC Strains
Source: PLoS One. 2014 Apr 17;9(4):e93804. doi: 10.1371/journal.pone.0093804 (PMC3990534; doi:10.1371/journal.pone.0093804)
Supplement: Table S2 — Sensitivity and resistance of different mutation sites in HIV-1 CRF_BC RT region to NRTIs. (DOC) [file pone.0093804.s002.doc]

Table S2 Sensitivity and resistance of different mutation sites in HIV-1 CRF_BC RT region to NRTIs

| Mutations | AZT | | D4T | | 3TC | | TDF | |
| --- | --- | --- | --- | --- | --- | --- | --- | --- |
|  | IC_50_^a^ | Fold change^c^ | IC_50_ | Fold change | IC_50_^a^ | Fold change | IC_50_ | Fold change |
| WT | 0.021±0.001 | - | 2.210±0.237 | - | 0.529±0.046 | - | 3.334±0.311 | - |
| **W88C** | 0.017±0.001 | 0.804 | 1.446±0.121 | 0.654 | 0.278±0.025 | 0.527 | 1.592±0.381 | 0.477 |
| **K101Q** | 0.013±0.002 | 0.633 | 1.381±0.222 | 0.625 | 0.216±0.036 | 0.409 | 2.515±0.473 | 0.754 |
| **I132L** | 0.014±0.001 | 0.681 | 0.732±0.089 | 0.331 | 0.092±0.014 | 0.175 | 0.714±0.105 | 0.214 |
| **T135L** | 0.021±0.001 | 0.992 | 1.130±0.051 | 0.511 | 0.278±0.047 | 0.526 | 2.611±0.381 | 0.783 |
| **T139K** | 0.033±0.005 | 1.563 | 1.567±0.163 | 0.709 | 0.287±0.044 | 0.543 | 3.006±0.426 | 0.902 |
| **T139R** | 0.017±0.003 | 0.837 | 1.695±0.171 | 0.767 | 0.297±0.034 | 0.561 | 2.791±0.495 | 0.837 |
| M184V | 0.012±0.000 | 0.586 | 1.020±0.183 | 0.461 | >10 | >18.914 | 1.402±0.109 | 0.421 |
| **H221Y** | 0.022±0.002 | 1.029 | 1.772±0.173 | 0.802 | 0.867±0.085 | 1.640 | 2.313±0.108 | 0.694 |
| **L228H** | 0.028±0.002 | 1.321 | 1.004±0.080 | 0.454 | 0.526±0.058 | 0.995 | 2.895±0.140 | 0.868 |

Note:^a^ Each sample was tested in triplicate, and each experiment was repeated twice. IC50 (μM) data are presented as means ± standard deviations. ^b^WT: CBJB257-14-1. ^c^Fold change was determined by calculating the ratio of IC_50_s for mutations and WT viruses.
